# A Prospective Assessment of the Effect of Aminophylline Therapy on Urine Output and Inflammation in Critically Ill Children

**DOI:** 10.3389/fped.2014.00059

**Published:** 2014-06-12

**Authors:** Robert F. Tamburro, Neal J. Thomas, Gary D. Ceneviva, Michael D. Dettorre, Gretchen L. Brummel, Steven E. Lucking

**Affiliations:** ^1^Department of Pediatrics, Division of Critical Care Medicine, Penn State Hershey Children’s Hospital, Pennsylvania State University College of Medicine, Hershey, PA, USA; ^2^Pharmacy Administration and Education Department, Milton S. Hershey Medical Center, Pennsylvania State University College of Medicine, Hershey, PA, USA

**Keywords:** aminophylline, respiratory function, inflammation, pediatrics, phosphodiesterase inhibition, adenosine receptor blockade, acute kidney injury

## Abstract

**Background:** Aminophylline, an established bronchodilator, is also purported to be an effective diuretic and anti-inflammatory agent. However, the data to support these contentions are scant. We conducted a prospective, open-label, single arm, single center study to assess the hypothesis that aminophylline increases urine output and decreases inflammation in critically ill children.

**Methods:** Children less than 18 years of age admitted to the pediatric intensive care unit who were prescribed aminophylline over a 24-h period were eligible for study. The use and dosing of aminophylline was independent of the study and was at the discretion of the clinical team. Data analyzed consisted of demographics, diagnoses, medications, and markers of pulmonary function, renal function, and inflammation. Data were collected at baseline and at 24-h after aminophylline initiation with primary outcomes of change in urine output and inflammatory cytokine concentrations.

**Results:** Thirty-five patients were studied. Urine output increased significantly with aminophylline use [median increase 0.5 mL/kg/h (IQR: −0.3, 1.3), *p* = 0.05] while blood urea nitrogen and creatinine concentrations remained unchanged. Among patients with elevated C-reactive protein concentrations, levels of both interleukin-6 (IL-6) and IL-10 decreased at 24 h of aminophylline therapy. There were no significant differences in pulmonary compliance or resistance among patients invasively ventilated at both time points. Side effects of aminophylline were detected in 7 of 35 patients.

**Conclusion:** Although no definitive conclusions can be drawn from this study, aminophylline may be a useful diuretic and effective anti-inflammatory medication in critically ill children. Given the incidence of side effects, the small sample size and the uncontrolled study design, further study is needed to inform the appropriate use of aminophylline in these children.

## Introduction

Aminophylline, the ethylenediamine salt of theophylline, is a well-established medication that promotes bronchodilatation by increasing the tissue concentrations of cyclic adenine monophosphate (cAMP) via phosphodiesterase inhibition (1). This pharmacological effect enables it to be useful in the treatment of a number of respiratory conditions including asthma (2). Despite this well documented efficacy, the advent of other bronchodilators that are effective, less prone to toxicity, and easier to administer and monitor has resulted in a dramatic decline in its use for reactive airway disease. However, in addition to its impact on air flow, aminophylline has been demonstrated to have other effects that may be beneficial to the critically ill child. It has been found in a variety of settings to be a useful diuretic and renoprotective agent (3–12). Moreover, various laboratory, animal, and small clinical studies have reported potent anti-inflammatory properties of aminophylline (13–19).

Given these potential renal and anti-inflammatory benefits, and its well-established benefit on bronchoconstriction, aminophylline may benefit a number of critically ill children with a variety of conditions. However, the data to support the use of aminophylline in this patient population is not well established. We undertook a prospective, open-label, and single arm study of the physiologic effects of aminophylline in a tertiary care pediatric intensive care unit (PICU). We hypothesized that aminophylline use would augment urine output and decrease inflammation in critically ill children.

## Materials and Methods

All patients less than 18 years of age admitted to the PICU who were prescribed aminophylline were screened for inclusion. Patients were excluded if they weighed less than 2.3 kg or if their initial hemoglobin concentration was less than 8 g/dL to minimize the risk associated with the extra blood draw required for the study. The Institutional Review Board of the Pennsylvania State University College of Medicine approved the protocol and informed consent was obtained for all patients enrolled.

**Figure 1:**
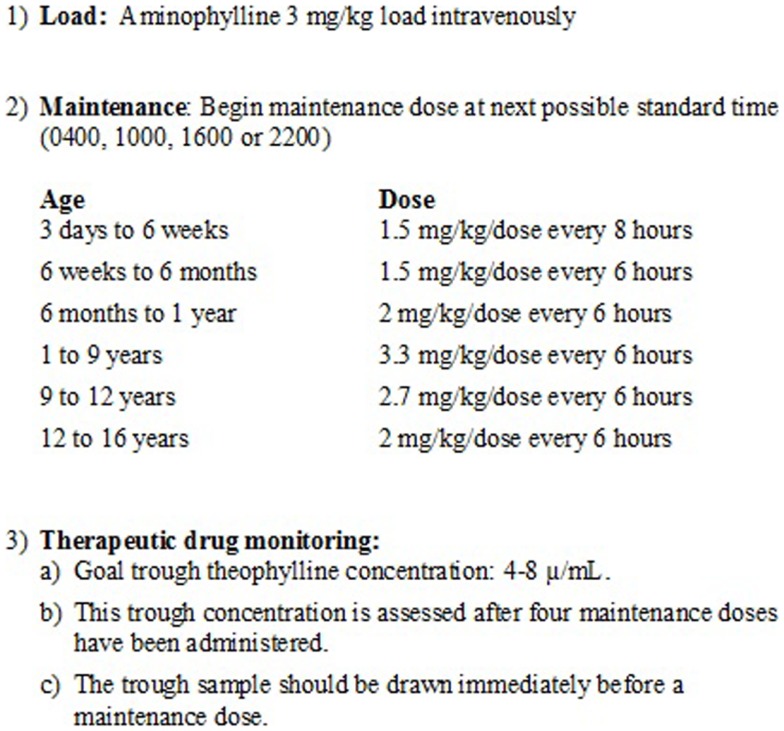
**Aminophylline dosing guidelines**. The figure depicts the standard intermittent dosing regimen used at the study center. This dosing regimen is based on institution-specific clinical experience. The goal trough theophylline concentration is 4–8 μg/mL. This trough concentration is assessed after four maintenance doses have been administered.

The use and dosing of aminophylline was independent of the study and was at the discretion of the clinical team. In our PICU, aminophylline administration is protocolized using an intermittent dosing regimen with a goal theophylline trough of 4–8 μg/mL (Figure 1). Trough concentrations are assessed every 24 h. When administered as a continuous infusion, primarily for severe bronchospasm (e.g., status asthmaticus), target theophylline concentrations are 10–20 μg/mL.

Data were collected at baseline prior to the administration of aminophylline, and then again, 24 h after the initiation of therapy. Collected data consisted of demographics, diagnoses, medications, and vital signs. In addition, data were obtained to assess the impact of aminophylline on renal function, inflammation, and pulmonary function as described below.

### Renal function

Data collected for the assessment of renal function consisted of serum blood urea nitrogen (BUN) and creatinine concentrations, diuretic use, and urine output. For the baseline determination of urine output, the previous 24 h prior to aminophylline administration was used. If urine was not collected for an entire 24 h prior to the aminophylline administration, the urine output for the entire time period prior to aminophylline administration was determined and standardized in terms of milliliters per kilogram per hour. Urine output was also determined by age group to assess if a relationship existed between aminophylline effect and age.

### Anti-inflammatory effects

Data collected for the assessment of inflammation consisted of white blood cell counts, C-reactive protein (CRP) concentrations, glucose concentrations, and anti-inflammatory medication use. Those patients with elevated CRP concentrations, defined *a priori* as values greater than 1.5 mg/dL at baseline, underwent measurements of interleukin-6 (IL-6), IL-8, IL-10, and tumor necrosis factor alpha (TNF-alpha). These samples, drawn at baseline (at the same time as the CRP level) and at 24-h, were immediately centrifuged for 10 min. After separation, the plasma was then stored at −70° centigrade. Cytokine analysis was performed on only those samples from patients found to have a CRP concentration greater than 1.5 mg/dL and it was performed utilizing multi-analyte profiling (MAP) technology (Myriad RBM, Inc., Austin, TX, USA). Although 90 different cytokine analyses were performed, it was decided *a priori* that only the four cytokines listed would be assessed in this study.

### Pulmonary function

Data collected relevant to the assessment of pulmonary function included blood gas results, pulse oximeter recordings, and ventilator settings. Additionally, airway resistance and compliance (both static and dynamic) were determined for intubated patients using the assessment tools of the Servo-I ventilator (Maquet Critical Care, Rastatt, Germany) with the patient sedated and not actively breathing over the ventilator.

### Statistical analysis

Descriptive statistics were performed for all variables; means, standard errors of the mean, medians, and interquartile ranges (IQR). There were two primary outcomes of interest; the effect of aminophylline on urine output and on concentrations of inflammatory cytokines with its impact on pulmonary function being a secondary outcome of interest. Consequently, a Wilcoxon Signed Rank test was performed to compare 24-h after aminophylline therapy values to baseline. To account for potential confounding effects of concomitant medications, total dosages were quantitated and four of the authors (Steven E. Lucking, Gary D. Ceneviva, Neal J. Thomas, Robert F. Tamburro) reviewed all medications and independently determined if there was an increase, a decrease, or no change in the dosing of other diuretics (for renal analysis) and anti-inflammatory medications (for inflammation analysis) across the two time points of the study. The authors were blinded to all outcomes of interest at the time of these determinations. If the authors were not in concordance, the case was discussed until all differences were resolved. A subset analysis was performed on only those patients who received the same or less of these other medications for their respective assessments. An alpha value of 0.05 defined statistical significance. Statistical analyses were performed using version 9 of the SAS statistical software program (SAS Institute, Inc., Cary, NC, USA).

## Results

**Figure 2:**
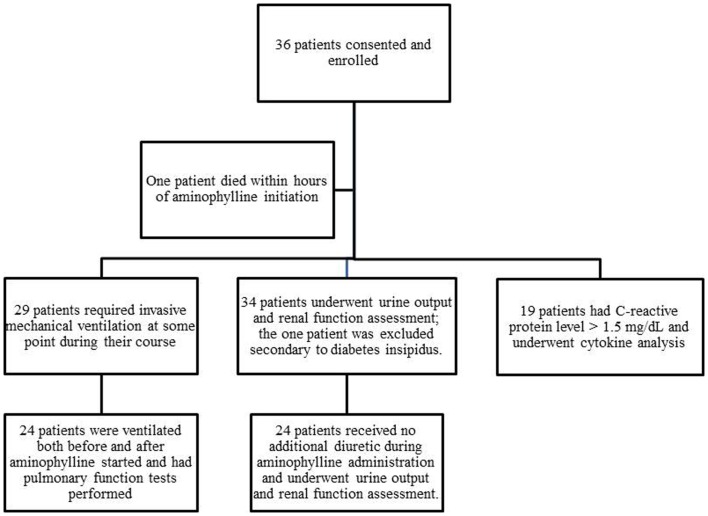
**Patients enrolled and assessed in various components of the study**. The figure depicts the total enrollment of patients into the trial and the number that underwent the various components of analysis.

Thirty-six patients were enrolled; 35 were available for analysis because one patient died within hours of enrollment (Figure 2). Ages ranged from 1 week to 18 years. The most common diagnoses included bronchiolitis (*n* = 9), cardiac disease (*n* = 6), and sepsis (*n* = 6) (Table 1).

**Table 1:** **Baseline data of entire study cohort**.

Variables	
**AGE (MONTHS)**	
Mean	51.5 ± 11.5
Median	14.8 [IQR: 1.6, 102.5]
**GENDER**	
Males	23 (66%)
Females	12 (34%)
**RACE/ETHNICITY**	
White	23 (66%)
Hispanic	10 (29%)
African American	2 (6%)
**PRIMARY DIAGNOSIS**	
Bronchiolitis	9 (26%)
Cardiac disease	6 (17%)
Sepsis	6 (17%)
Pneumonia	4 (11%)
Bronchopulmonary dysplasia	3 (9%)
Near drowning	2 (6%)
Neuromuscular disease	2 (6%)
Tracheal ring	1 (3%)
Thoracic mass	1 (3%)
Status asthmaticus	1 (3%)
**CHEST RADIOGRAPHS**	
Bilateral disease	27 (77%)
Unilateral disease	3 (9%)
Clear	5 (14%)
**BASELINE RESPIRATORY SUPPORT**	
Invasive ventilation	28 (80%)
BiLevel Positive Airway Pressure (BiPAP)	3 (9%)
Nasal cannula	2 (6%)
High flow nasal cannula (VapoTherm^®^)	1 (3%)
Face mask	1 (3%)

### Renal function

Thirty-four patients had pre- and post-aminophylline urine output assessed; one patient was excluded from this analysis because of concomitant diabetes insipidus. Among these 34 patients, the urine output increased from a median of 3.5 [IQR: 2.0, 5.0] at baseline to 4.2 [IQR: 2.7, 5.6] at 24-h with a median increase of 1.0 mL/kg/h ([IQR: −0.1, 1.5], *p* = 0.0004) (Table 2). The BUN concentrations remained unchanged between baseline and 24-h (median increase 1.0 mg/dL [IQR: 0.0, 4.0], *p* = 0.16). There was also no change in the creatinine concentrations (median increase 0.02 mg/dL [IQR: −0.07, 0.07], *p* = 0.99) (Table 2).

**Table 2:** **Assessment of renal variables before and after 24 h of aminophylline**.

Variable	Baseline value	24-h value	*p* Value
**ENTIRE COHORT (*n* = 34)**			
Urine output^a^	3.6 ± 0.4 (3.5 [2.0, 5.0])	4.7 ± 0.5 (4.2 [2.7, 5.6])	0.0004
BUN^b^	20.7 ± 3.1 (13.5 [8.0, 27.0])	22.5 ± 3.9 (15.0 [9.0, 25.0])	0.16
Creatinine^b^	0.70 ± 0.15 (0.38 [0.27, 0.55])	0.75 ± 0.19 (0.34 [0.30, 0.55])	0.99
**SUBSET THAT RECEIVED THE SAME OR LESS CONCOMITANT DIURETICS DURING THE 24 h AFTER THE START OF AMINOPHYLLINE (*n****=*** 24)**			
Urine output^a^	3.6 ± 0.5 (3.4 [1.9, 5.1])	4.4 ± 0.6 (3.5 [2.3, 5.5])	0.05
BUN^b^	24.0 ± 4.0 (14.5 [10.5, 33.5])	26.7 ± 5.1 (15.0 [10.0, 28.0])	0.15
Creatinine^b^	0.75 ± 0.18 (0.41 [0.32, 0.69])	0.81 ± 0.24 (0.37 [0.31, 0.66])	0.30

*Both the mean plus/minus the standard error of the mean as well as the median (in parentheses) with the corresponding interquartile range [in brackets] are displayed for each value at each time point*.

*The *p value* was determined by the difference of the two values for each patient using the Wilcoxon Signed Rank test and was based on only the patients who had values recorded at both time points*.

*^a^ Urine output is reported in terms of milliliters per kilogram per hour*.

*^b^ The units for BUN and creatinine are milligrams per deciliter*.

Thirty-one patients received concomitant diuretics during the study period. Ten patients received increased diuretic therapy during the 24 h of aminophylline therapy. Consequently, urine output was assessed among the 24 patients that received the same or less concomitant diuretics during the 24 h after the start of aminophylline as during the previous 24 h. Within this subset, the urine output significantly increased with a median increase of 0.5 mL/kg/h per patient ([IQR: −0.3, 1.3], *p* = 0.05) (Table 2). Fifteen of these 24 patients (63%) experienced an increased urine output with aminophylline use. There was no change in BUN concentrations among this cohort (median increase 1.0 [IQR: 0.0, 4.0], *p* = 0.15) nor in creatinine values (median decrease 0.03 [IQR: −0.08, 0.05], *p* = 0.30) (Table 2).

**Figure 3:**
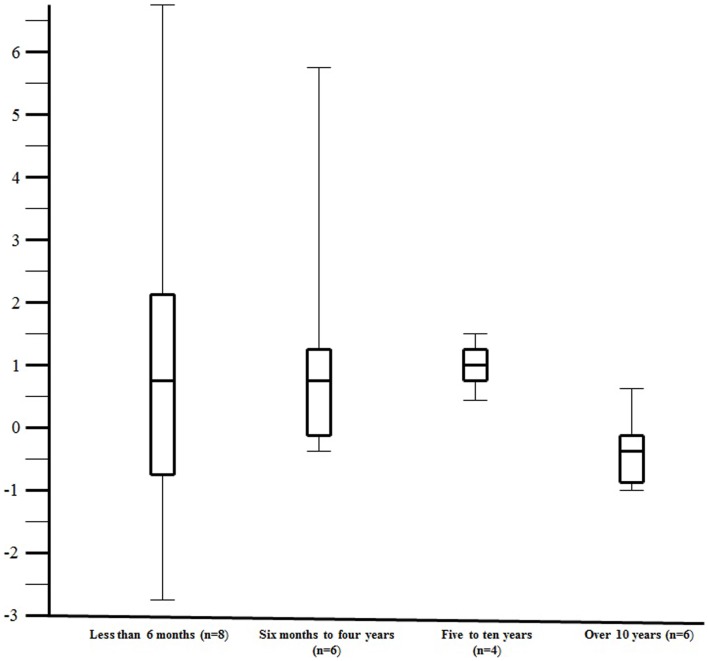
**The change in urine output (milliliters per kilogram per hour) over the 24 h of aminophylline therapy by age group**. The figure illustrates the change in urine output (milliliters per kilogram per hour) over the 24 h of aminophylline therapy by age group. The change in urine output (milliliters per kilogram per hour) over the 24 h of aminophylline therapy is plotted along the *y*-axis. The *x*-axis contains the four age groups divided in approximately equal groups. The number of study patients assessed in age group is depicted by the “*n* =” within the parentheses. The center line within the “box” reflects the median while the ends of the “box” reflect the 25th and 75th percentile, respectively. The “whiskers” extending from the box reflect the entire range of values.

In an attempt to determine if there was an effect of age on response to aminophylline, urine output was assessed by age group among the 24 patients that received the same or less concomitant diuretics during the 24 h after the start of aminophylline. Although the numbers are small in each age group, aminophylline appeared to be more effective in the children less than 10 years of age as compared to those over 10 years of age (Figure 3, median change in urine output 1.0 [IQR: −0.1, 1.5] versus −0.2 [IQR: −0.7, 0.1], respectively, *p* = 0.05).

### Anti-inflammatory effects

There was no change in white blood cell count or glucose concentration between the two time points and comparable results were obtained for these parameters for both the entire cohort and for the subset with elevated CRP concentrations (Table 3). Nineteen patients had CRP concentrations greater than 1.5 mg/dL and underwent cytokine analysis. For these patients, baseline CRP values ranged from 1.7 to >27 mg/dL (median 9.0 [IQR: 4.1, 24.3]. The median decrease in IL-6 concentrations between baseline and 24 h was 17.6 pg/mL [IQR: −5.1, −43.7], *p* < 0.0001) (Table 3). Excluding the three patients who had an increase in their steroid or other anti-inflammatory medications during the 24 h after the start of aminophylline, yielded comparable results (median decrease 19.0 [IQR: −8.6, −57.7], *p* < 0.0001) (Table 3). Baseline IL-10 concentrations also differed from 24-h concentrations; the median decrease in IL-10 concentrations was 15.0 ([IQR: −3.0, −33.0], *p* = 0.001) (Table 3). When the three patients who had an increase in their anti-inflammatory medications during the 24 h after aminophylline initiation were excluded, the median decrease in IL-10 concentrations of 12.0 (IQR: −2.5, −41.0) was comparable to the entire cohort and remained statistically significant (*p* = 0.009) (Table 3). Baseline IL-8 concentrations also differed from 24-h concentrations; (median decrease 14.0 [IQR: −2.0, −36.0], *p* = 0.05) (Table 3). However, when the three patients who had an increase in their concomitant anti-inflammatory medications were excluded, the median decrease in IL-8 concentrations of 13.2 (IQR: −2.2, −41.2) only trended toward significance (*p* = 0.09) (Table 3). TNF-alpha concentrations did not change over the 24 h study period (Table 3).

**Table 3:** **Assessment of inflammatory parameters before and after 24 h of aminophylline**.

Variable	Baseline value	24-h value	*p* Value
**SUBSET THAT HAD AN ELEVATED C-REACTIVE PROTEIN AT BASELINE (*n* = 19)**			
White blood cell^a^	11.4 ± 2.0 (9.4 [7.5, 11.2])	10.9 ± 1.6 (10.0 [6.5, 12.8])	0.76
Glucose^b^	125.6 ± 8.7 (119 [97, 145])	127.7 ± 7.9 (121 [106, 143])	0.99
IL-6^c^	156.1 ± 89.7 (27.0 [9.4, 172.0])	37.3 ± 13.9 (9.4 [4.3, 20.0])	<0.0001
IL-8^c^	199.7 ± 69.3 (89 [51, 141])	202.7 ± 98.1 (64 [34, 99])	0.05
IL-10^c^	211.5 ± 113.1 (40.0 [20.0, 58.0])	72.9 ± 34.6 (21.0 [14.0, 33.0])	0.001
TNF-alpha^c^	15.4 ± 3.1 (12.0 [7.5, 17.0])	11.0 ± 1.6 (8.5 [6.8, 12.0])	0.26
**SUBSET OF ABOVE COHORT THAT RECEIVED THE SAME OR LESS CONCOMITANT ANTI-INFLAMMATORY MEDICATIONS DURING THE 24 h AFTER THE START OF AMINOPHYLLINE (*n* = 16)**			
IL-6^c^	180.2 ± 105.9 (27.0 [12.7, 183.0])	42.4 ± 16.3 (7.6 [4.3, 56.5])	<0.0001
IL-8^c^	218.7 ± 81.7 (85.5 [49.5, 164.0])	225.4 ± 116.1 (61.0 [33.5, 157.0])	0.09
IL-10^c^	106.1 ± 53.5 (33.0 [16.5, 48.5])	78.7 ± 41.0 (17.0 [13.5, 30.0])	0.009
TNF-alpha^c^	12.8 ± 2.5 (10.5 [6.8, 14.0])	10.8 ± 1.9 (8.0 [6.5, 12.0])	0.26

*Both the mean plus/minus the standard error of the mean as well as the median (in parentheses) with the corresponding interquartile range [in brackets] are displayed for each value at each time point*.

*The *p value* was determined by the difference of the two values for each patient using the Wilcoxon Signed Rank test and was based on only the patients who had values recorded at both time points*.

*^a^ White blood cell count expressed in terms of 1000 cells/μL*.

*^b^ Blood glucose levels expressed in units of milligrams per deciliter*.

*^c^ Cytokine levels expressed in units of picograms per milliliter*.

### Pulmonary function

Twenty-eight patients received invasive mechanical ventilation at baseline; three others required non-invasive bilateral positive airway pressure. Twenty-seven (77%) patients had bilateral lung involvement based on their chest radiograph (Table 1).

Twenty-four patients were invasively ventilated at both time points (prior to aminophylline and at 24-h after the start of aminophylline) and underwent assessment of respiratory function. Among this group, there were no statistically significant differences in static compliance (median change 0.40 mL/cm H_2_O [IQR: −0.45, 0.80], *p* = 0.33) or airway resistance (median change 0.5 cm H_2_O/L/s [IQR: −35.5, 4.5], *p* = 0.29) between the two time points (Table 4). There was an increase in dynamic compliance that approached statistical significance (median change 0.40 [IQR: 0.0, 0.60], *p* = 0.08).

**Table 4:** **Assessment of pulmonary variables before and after 24 h of aminophylline**.

Entire cohort intubated and ventilated at both time points (*n* = 24)			
Variable	Baseline value	24-h value	*p* Value
Static compliance^a^	9.5 ± 2.1 (4.4 [2.8, 14.6])	12.4 ± 3.0 (5.3 [3.0, 16.4])	0.33
Dynamic compliance^a^	8.6 ± 1.9 (3.7 [2.3, 14.7])	11.3 ± 3.0 (4.0 [2.3, 18.4])	0.08
Resistance^b^	106.1 ± 13.8 (100 [34, 137])	102.3 ± 19.5 (79 [34, 142])	0.29
pH	7.42 ± 0.01 (7.43 [7.39, 7.47])	7.46 ± 0.01 (7.46 [7.42, 7.49])	0.002
SF ratio^c^	240 ± 14 (232.5 [190, 325])	248 ± 15 (245 [194, 323.3])	0.17

*Both the mean plus/minus the standard error of the mean as well as the median (in parentheses) with the corresponding interquartile range [in brackets] are displayed for each value at each time point*.

*The *p value* was determined by the difference of the two values for each patient using the Wilcoxon Signed Rank test and was based on only the patients who had values recorded at both time points*.

*^a^ The units for compliance are milliliters per centimeter H_2_O*.

*^b^ The units for resistance are centimeter H_2_O/L/s*.

*^c^ SF ratio signifies the oxygen saturation to the fraction of inspired oxygen ratio*.

### Drug concentrations and side effects

Aminophylline was administered as a continuous infusion in 3 of the 35 (9%) patients; the others received it via intermittent dosing. Among the three patients who received aminophylline as a continuous infusion, the 24-h serum theophylline concentrations were 10.5, 12.1, and 13.9 μg/mL. The median 24-h serum trough theophylline concentration for the patients who received intermittent dosing was 3.7 with a range of 1.1–9.4 μg/mL [IQR: 2.8, 5.8].

Side effects were identified in 7 of the 35 patients (20%) (all receiving intermittent dosing) during their intensive care course, primarily occurring *after* the defined study period (i.e., after the initial 24 h of aminophylline therapy) (Table 5). All patients completed at least 24 h of therapy. Aminophylline was ultimately discontinued in the seven patients; again, none within the first 24 h of therapy, but three within the first 48 h. Identified side effects included agitation, increased nasogastric output, cardiac ectopy, and tachydysrhythmias (Table 5). Four patients developed dysrhythmias including supraventricular tachycardia, ventricular tachycardia, and pre-mature atrial contractions. Given the critical condition of these children, other explanations existed for the dysrhythmias. Despite the occurrence of these tachydysrhythmias, the baseline median heart rate for the entire cohort was 138 beats per minute [IQR: 127, 151] and remained essentially unchanged at 24 h (median 141 [IQR: 126, 154], *p* = 0.74).

**Table 5:** **Characteristics of patients with documented side effects**.

Diagnoses	Side effect	Day	Associated clinical condition
S/P cardiopulmonary arrest	Increased NG output	3	Cardiomyopathy
			Hypoxic ischemic encephalopathy
Pneumonia	PVCs/bradycardia	2	Pulmonary hypertension
Obstructive sleep apnea	Sinus arrhythmia		PVCs resolved with potassium and magnesium therapy
Severe septic shock AML	SVT/VT Junctional tachycardia	3	Hypotensive, febrile, oliguric, acidotic; died 3 days later
ASD repaired 11 years ago
Severe septic shock Necrotizing enterocolitis	SVT	3	SVT occurred following albuterol and aminophylline dose; resolved with vagal maneuver
Pneumonia	Sinus tachycardia	2	Heart rate in the 190–210
SMA type 1
RSV bronchiolitis TOF (previously repaired)	PACs	2	PACs resolved with potassium
RSV bronchiolitis	Agitation	7	

*NG, nasogastric; PVCs, premature ventricular contractions; SVT, supraventricular tachycardia; VT, ventricular tachycardia; AML, acute myelocytic leukemia; ASD, atrioventricular septal defect; SMA, spinal muscular atrophy; RSV, respiratory syncytial; PACs, premature atrial contractions; TOF, Tetralogy of Fallot*.

## Discussion

In this prospective, open-label, single arm, single center study, the use of aminophylline in critically ill children appeared to be associated with an increase in urine output and a decrease in cytokines of paramount importance to the inflammatory process.

The use of aminophylline for renal protection from acute kidney injury (AKI) is not novel. The efficacy of aminophylline for renal protection has been reported in cisplatin- (3), tacrolimus- (4), and contrast-induced (5, 6) AKI, in animal models of kidney transplantation (20), and in the vasomotor nephropathy of very pre-term newborns (21). However, its use in critically ill infants and children is not well described. Bell reported a small case series of 10 critically ill children who had a significant increase in urine output with theophylline administration (7). However, in that report, there was no report of BUN or creatinine concentrations, and the concomitant use of low dose dopamine confounded the interpretation and the general applicability of the results. In another small retrospective case series, the administration of aminophylline to five neonates appeared to be associated with an increase in urine output; however, the lack of a bladder catheter complicated interpretation of the study results (8). Lynch reported a retrospective case series of 13 neonates with non-oliguric AKI and described an improvement in renal function indices with aminophylline (9). However, in that publication, urine outputs were not reported, and many of the subjects were receiving concomitant caffeine, which shares similar properties of aminophylline (9). In a relatively recent retrospective study of nine children with AKI, aminophylline use resulted in improved urine flow rates, stable creatinine concentrations, and a reduced requirement for dialysis (10). Although each of these studies supports a beneficial effect of aminophylline in renal injury, all have significant limitations. The findings of this current report suggest in a prospective manner, with a relatively large sample size, that this therapeutic intervention can increase urine output while not increasing BUN or creatinine concentrations suggesting improved renal perfusion. The results also suggest that this response may be age dependent; however, given the small subgroup sample sizes, this finding can, at best, be used solely to inform future study design.

The purported renal benefits of aminophylline have been attributed to two mechanisms: adenosine receptor blockade at low dosage and type IV phosphodiesterase inhibition at high levels. Adenosine is the putative mediator of tubuloglomerular feedback (22). When the tubule is exposed to increased solute, energy depletion occurs, and adenosine is released. The released adenosine triggers pre-glomerular vasoconstriction thereby limiting solute flow and, in theory, restoring energy balance. In addition to adenosine receptor blockade, aminophylline-induced phosphodiesterase inhibition may prevent the hydrolysis of cAMP, which is important in the mediation of renal blood flow as it promotes renal vasodilatation (23). Both of these mechanisms likely contribute to the improved renal blood flow attributed to aminophylline.

In addition to its effect on the kidneys, significant reductions in cytokines IL-6, IL-10, and to a lesser degree IL-8, were detected following 24 h of aminophylline therapy. Aminophylline has been found to influence the immune response in other reports. Studies have demonstrated that aminophylline can suppress the release of eosinophil cationic protein (13), induce apoptosis of activated eosinophils (13), inhibit eosinophil chemotaxis in response to multiple stimuli (14), and suppress mitogen-induced secretion of pro-inflammatory cytokines from human peripheral blood lymphocytes (15). In addition, Luo demonstrated significantly lower concentrations of IL-8 and TNF-alpha, and significantly higher concentrations of IL-10 in adult patients receiving aminophylline during cardiopulmonary bypass for valve replacement (24). Although their results differ from ours, they observed a dramatic surge in cytokines after baseline with the use of cardiopulmonary bypass confounding any comparison.

The mechanism by which aminophylline suppresses inflammation is not entirely established. It has been demonstrated that its anti-inflammatory effect can occur at doses significantly smaller than typical bronchodilator doses (i.e., concentrations lower than those needed to produce phosphodiesterase inhibition) (25). Theophylline concentrations on the order of those reported in this study have been found to suppress endotoxin-induced TNF-alpha production by monocytes *in vivo* (26). Thus, it has been postulated that the anti-inflammatory effects may be due to mechanisms other than phosphodiesterase inhibition. Adenosine receptor antagonism (27), an increase in histone deacetylase activity (28), and/or enhanced messenger RNA and protein levels of peroxisomal proliferator-activated receptor-gamma (PPAR-gamma) expression (29) have all been reported as potential explanations.

Additionally, aminophylline exerts a number of beneficial effects on pulmonary function via phosphodiesterase inhibition, adenosine receptor antagonism, and increased catecholamine release. In addition to bronchodilation, it has also been found to decrease mucosal edema (30) and excessive secretions (31), enhance respiratory muscle contractility (32) and stimulate the medullary respiratory center (33). In this study, aminophylline was found to have little effect on airway resistance or respiratory compliance. The lack of effect on airway resistance could seemingly be explained by the small and heterogeneous sample. However, other physiologic explanations exist. For example, a large number of patients in the trial had no evidence of increased airway resistance. If bronchospasm is absent, the effects of aminophylline on air flow and respiratory mechanics may be minimal (34). In addition, aminophylline-induced-bronchodilation is proportional to the log of the plasma theophylline concentration over a range of 5–25 μg/mL (35). It is plausible that the relatively low levels of theophylline attained in this trial were insufficient to induce significant bronchodilation. Moreover, the ability of aminophylline to produce bronchodilation is most likely secondary to phosphodiesterase inhibition and varies from patient to patient (36). In therapeutic doses, aminophylline can only inhibit between 5 and 20% of total phosphodiesterase activity in human lungs (37).

Any conclusions drawn from this report regarding aminophylline use must be tempered by the obvious study limitations, and the potential for significant side effects. First, the uncontrolled design of this study confounds any definitive interpretation of the results. It is impossible to determine if the improvement in urine output and the reductions reported in cytokine concentrations were truly the result of aminophylline therapy or the natural course of disease. Although the study patients consisted of a diverse group of diagnoses at variable stages of their disease course, a true effect of aminophylline on urine output and inflammation can only be discerned with the use of an appropriate control group. Second, the study is limited by the small sample size and the diverse patient population. Although the small sample size clearly limits the analysis of the study data and its extrapolation, it is important to note that this study population is larger than any of the previous reports assessing the diuretic and anti-inflammatory properties of aminophylline in critically ill infants and children. Moreover, an analysis by age group was conducted to minimize some of the heterogeneity associated with the large age range of study patients. Finally, the apparent benefits of aminophylline therapy for this patient population must be balanced against the potential for adverse sequelae associated with its use. Although pulmonary, renal and inflammatory effects were assessed for only 24 h, side effects beyond the 24-h study period were reported for completeness.

In light of these clear and acknowledged weaknesses and concerns, the results of this study cannot be considered definitive and the use of aminophylline cannot be routinely recommended for the purposes of augmenting urine output and/or decreasing inflammation in critically ill children. Current national guidelines only suggest that a single dose of theophylline may be administered to neonates with severe perinatal asphyxia at risk for AKI (38). The findings of this study suggest that aminophylline may be of benefit in other populations. A recent publication suggests that it may improve renal function in children with AKI and congenital or acquired cardiac disease admitted to the cardiovascular intensive care unit (39). Given the surge of publications implicating positive fluid balance with worse outcomes in adults (40) and children (41, 42), and the established deleterious consequences of uncontrolled inflammation in the critically ill, the use of aminophylline has the potential to be of much benefit for these children. The results of this trial should serve to stimulate and inform well designed, randomized, controlled trials that may provide the needed insight to direct the appropriate use of aminophylline in critically ill children balancing potential benefit against the risk of toxicity.

## Conflict of Interest Statement

Neal Thomas serves as a consultant for Discovery Laboratories and receives funding for his efforts. Gretchen Brummel works as a consultant for Lexicomp, a “Pharma-free” drug information vendor, and receives pay for her services. She is a former Lexicomp employee. Robert Tamburro and Neal Thomas receive funding including salary support from the United States Food and Drug Administration Office of Orphan Product Development Grant Program. We have no other conflicts to disclose.
